# Crystal structure of 2-amino-4-(4-meth­oxy­phen­yl)-4*H*-benzo[*g*]chromene-3-carbo­nitrile

**DOI:** 10.1107/S205698901502280X

**Published:** 2015-12-06

**Authors:** Joel T. Mague, Shaaban K. Mohamed, Mehmet Akkurt, Sabry H. H. Younes, Mustafa R. Albayati

**Affiliations:** a Department of Chemistry, Tulane University, New Orleans, LA 70118, USA; b Chemistry and Environmental Division, Manchester Metropolitan University, Manchester M1 5GD, England; c Chemistry Department, Faculty of Science, Minia University, 61519 El-Minia, Egypt; d Department of Physics, Faculty of Sciences, Erciyes University, 38039 Kayseri, Turkey; e Chemistry Department, Faculty of Science, Sohag University, 82524 Sohag, Egypt; f Kirkuk University, College of Science, Department of Chemistry, Kirkuk, Iraq

**Keywords:** crystal structure, chromenes, benzo­pyrans, 2-amino-3-cyano-4*H*-chromene derivatives, 4*H*-chromene and fused 4*H*-chromene derivatives, hydrogen bonding

## Abstract

In the title compound, C_21_H_16_N_2_O_2_, the naphthalene fragment is twisted slightly, as indicated by the dihedral angle of 3.2 (2)° between the two six-membered rings. The pendant 4-meth­oxy­phenyl ring makes a dihedral angle of 86.08 (6)° with the central six-membered ring of the 4*H*-benzo[*g*]chromene ring system. In the crystal, mol­ecules are linked by pairs of N—H⋯N hydrogen bonds, forming inversion dimers which are linked into chains propagating in the *b*-axis direction by N—H⋯O hydrogen bonds.

## Related literature   

For the chemical and pharmacological properties of 4*H*-chromene and fused 4*H*-chromene derivatives, see: Bonsignore *et al.* (1993 ▸); Martínez-Grau & Marco (1997 ▸); Abd-El-Aziz *et al.* (2007 ▸); Sabry *et al.* (2011 ▸). For the synthesis and biological activities of 2-amino-3-cyano-4*H*-chromene derivatives, see: Kemnitzer *et al.* (2005 ▸); Patil *et al.* (2012 ▸); Kumar *et al.* (2009 ▸).
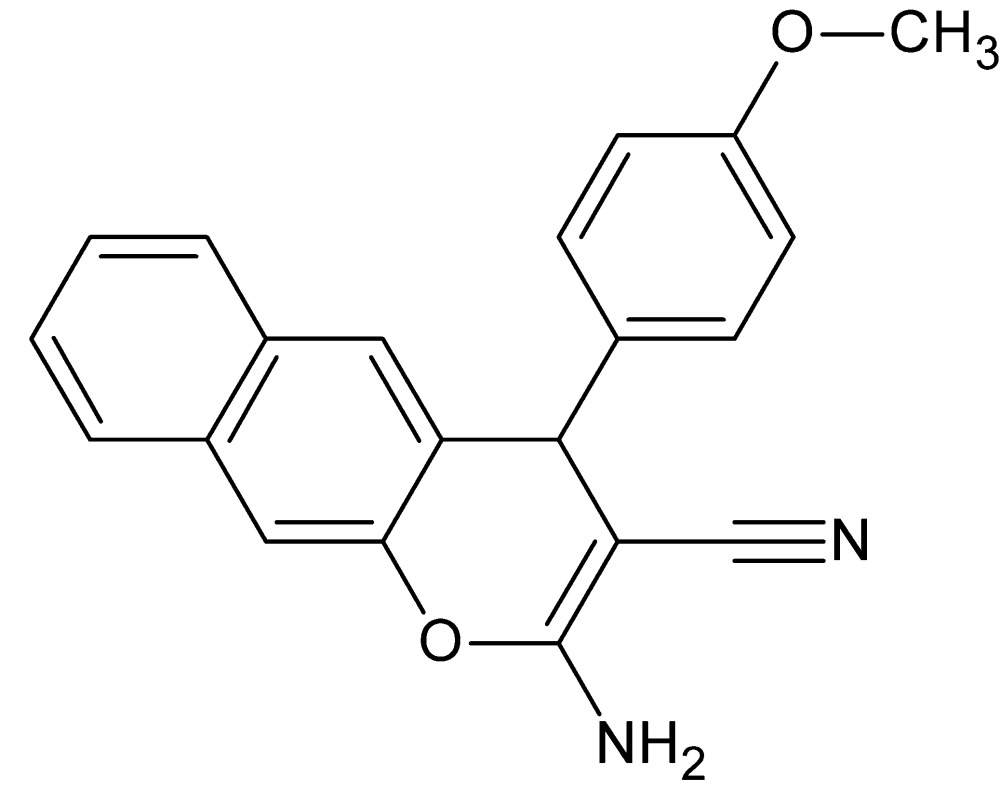


## Experimental   

### Crystal data  

C_21_H_16_N_2_O_2_
*M* *_r_* = 328.36
Triclinic, 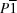

*a* = 6.3833 (2) Å
*b* = 10.6009 (3) Å
*c* = 13.0915 (4) Å
α = 108.823 (2)°
β = 95.906 (2)°
γ = 97.467 (2)°
*V* = 821.44 (4) Å^3^
*Z* = 2
Cu *K*α radiation
μ = 0.69 mm^−1^
*T* = 150 K
0.26 × 0.20 × 0.02 mm

### Data collection  

Bruker D8 VENTURE PHOTON 100 CMOS diffractometer
Absorption correction: multi-scan (*SADABS*; Bruker, 2015 ▸) *T*_min_ = 0.78, *T*_max_ = 0.99
6215 measured reflections
3039 independent reflections
2074 reflections with *I* > 2σ(*I*)
*R*_int_ = 0.048

### Refinement  

*R*[*F* ^2^ > 2σ(*F* ^2^)] = 0.057
*wR*(*F* ^2^) = 0.151
*S* = 1.03
3039 reflections
235 parameters
H atoms treated by a mixture of independent and constrained refinement
Δρ_max_ = 0.18 e Å^−3^
Δρ_min_ = −0.22 e Å^−3^

### 

Data collection: *APEX2* (Bruker, 2015 ▸); cell refinement: *SAINT* (Bruker, 2015 ▸); data reduction: *SAINT*; program(s) used to solve structure: *SHELXT* (Sheldrick, 2015*a* ▸); program(s) used to refine structure: *SHELXL2014* (Sheldrick, 2015*b* ▸); molecular graphics: *DIAMOND* (Brandenburg & Putz, 2012 ▸); software used to prepare material for publication: *SHELXTL* (Sheldrick, 2008 ▸).

## Supplementary Material

Crystal structure: contains datablock(s) global, I. DOI: 10.1107/S205698901502280X/su5248sup1.cif

Structure factors: contains datablock(s) I. DOI: 10.1107/S205698901502280X/su5248Isup2.hkl

Supporting information file. DOI: 10.1107/S205698901502280X/su5248Isup3.cml

**Fig. 1.**
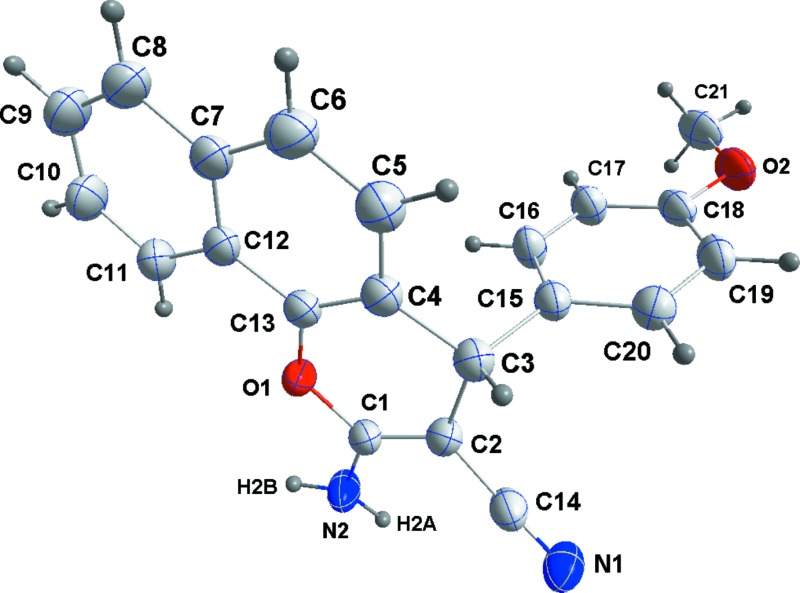
The molecular structure of the title compound with the labeling scheme and 50% probability displacement ellipsoids.

**Fig. 2.**
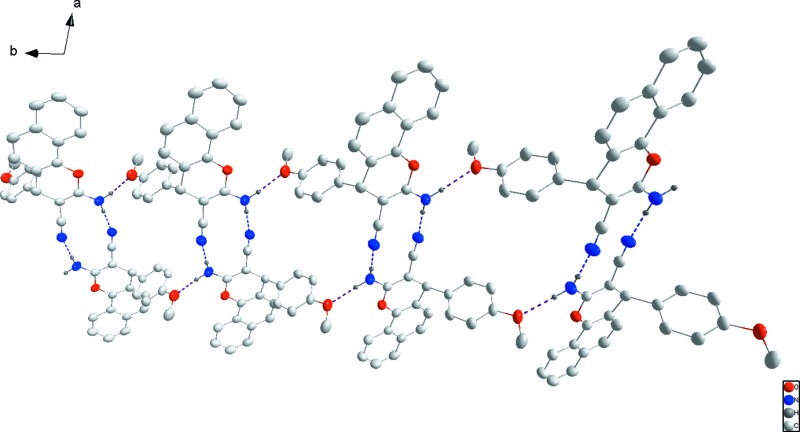
View along the c axis of one hydrogen-bonded layer. The N—H···N and N—H···O hydrogen bonds (see Table 1) are shown as blue and purple dotted lines, respectively.

. DOI: 10.1107/S205698901502280X/su5248fig1.tif
The mol­ecular structure of the title compound with the labeling scheme and 50% probability displacement ellipsoids.

c . DOI: 10.1107/S205698901502280X/su5248fig2.tif
View along the *c* axis of one hydrogen-bonded layer. The N—H⋯N and N—H⋯O hydrogen bonds (see Table 1) are shown as blue and purple dotted lines, respectively.

**Fig. 3.**
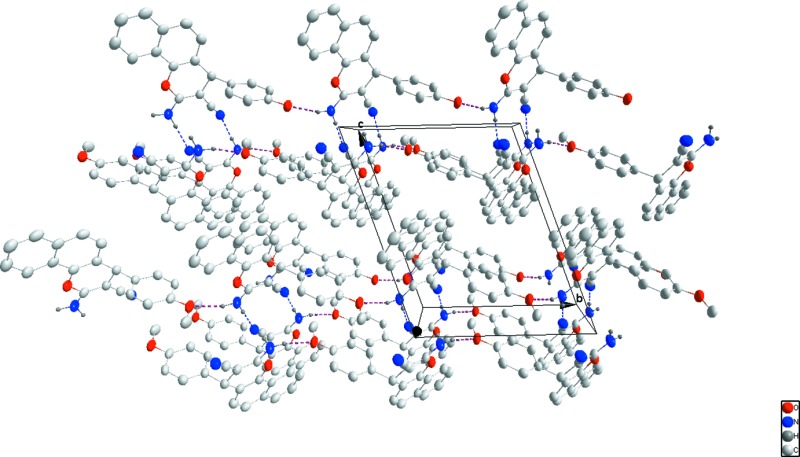
Crystal packing viewed along the c axis, with the N—H···N and N—H···O hydrogen bonds (see Table 1) shown as blue and purple dotted lines, respectively.

. DOI: 10.1107/S205698901502280X/su5248fig3.tif
Crystal packing viewed along the c axis, with the N—H⋯N and N—H⋯O hydrogen bonds (see Table 1) shown as blue and purple dotted lines, respectively.

CCDC reference: 1439459

Additional supporting information:  crystallographic information; 3D view; checkCIF report

## Figures and Tables

**Table 1:** Hydrogen-bond geometry (Å, °)

*D*—H⋯*A*	*D*—H	H⋯*A*	*D*⋯*A*	*D*—H⋯*A*
N2—H2*A*⋯N1^i^	0.96 (3)	2.03 (3)	2.995 (3)	178 (3)
N2—H2*B*⋯O2^ii^	0.95 (3)	2.10 (3)	3.028 (3)	166 (2)

Symmetry codes: (i) 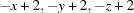; (ii) 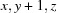.

## supplementary crystallographic information

### S1. Structural commentary

Fused benzo-4*H*-pyran, namely, 4*H*-chromene moiety, is the key building block of many oxygen-containing heterocyclic natural products whose pharmacological and biological activity such as anti-tumor, anti-oxidant, anti-bacterial, anti-viral, anti-fungal, hypotensive, anti-coagulant, anti-leishmanial, diuretic, and anti-allergenic activities (Bonsignore *et al.*, 1993; Martínez-Grau & Marco, 1997; Abd-El-Aziz *et al.*, 2007; Sabry *et al.*, 2011). Moreover, 2-amino-3-cyano-4*H*-chromene derivatives have been also used as anti-cancers, inhibitors of insulin-regulated amino­peptidase (IRAP) for enhancing memory and learning functions and anti-bacterial agents (Kemnitzer *et al.*, 2005; Patil *et al.*, 2012; Kumar *et al.*, 2009). In continuation of our inter­est in the chemical and pharmacological properties of 4*H*-chromene and fused 4*H*-chromene derivatives, we herein report on the synthesis and crystal structure of the title compound.

In the title compound, Fig. 1, the naphthalene fragment is slightly twisted as indicated by the dihedral angle of 3.2 (2)° between the two 6-membered rings. The pendant 4-meth­oxy­phenyl ring makes a dihedral angle of 86.08 (6)° with the (C4—C7/C12/C13) ring. The heterocyclic ring (O1/C1—C4/C13) can best be described as having an envelope conformation, with atom C3 as the flap, and with pucking parameters of Q = 0.099 (2) Å, θ = 109.8 (12)° and φ = 6.5 (14)°.

In the crystal, pairwise N2—H2A···N1^i^ hydrogen bonds form inversion dimers which are linked into chains running along the *b* axis direction (Fig. 2 and Table 1). The overall packing of these units in the crystal is illustrated in Fig. 3.

### S2. Synthesis and crystallization

To a solution of 4-meth­oxy­benzyl­idene-malono­nitrile (1 mmol, 180 mg) in 10 ml of ethanol was added 4- 1-naphthol (1 mmol, 144 mg) in the presence of few catalytic drops of piperedine and the temperature was adjusted at 353 K for 1 h. A solid product was obtained on cooling, collected by filtration and recrystallized from ethanol to afford colorless plate-like crystals suitable for X-ray diffraction analysis.

### S3. Refinement

The NH_2_ H-atoms were located in a difference Fourier map and freely refined. The C-bound H atoms were placed in calculated positions (C—H = 0.95 − 1.00 Å) and included as riding contributions with *U*_iso_(H) = 1.5*U*_eq_(*C*-methyl) and 1.2*U*_eq_(C) for other H atoms.

### Crystal data

**Table:**

C_21_H_16_N_2_O_2_	*Z* = 2
*M**_r_* = 328.36	*F*(000) = 344
Triclinic, *P*1	*D*_x_ = 1.328 Mg m^−^^3^
*a* = 6.3833 (2) Å	Cu *K*α radiation, λ = 1.54178 Å
*b* = 10.6009 (3) Å	Cell parameters from 3044 reflections
*c* = 13.0915 (4) Å	θ = 3.6–72.4°
α = 108.823 (2)°	µ = 0.69 mm^−^^1^
β = 95.906 (2)°	*T* = 150 K
γ = 97.467 (2)°	Plate, colourless
*V* = 821.44 (4) Å^3^	0.26 × 0.20 × 0.02 mm

### Data collection

**Table:**

Bruker D8 VENTURE PHOTON 100 CMOS diffractometer	3039 independent reflections
Radiation source: INCOATEC IµS micro–focus source	2074 reflections with *I* > 2σ(*I*)
Mirror monochromator	*R*_int_ = 0.048
Detector resolution: 10.4167 pixels mm^-1^	θ_max_ = 72.4°, θ_min_ = 3.6°
ω scans	*h* = −7→6
Absorption correction: multi-scan (*SADABS*; Bruker, 2015)	*k* = −12→13
*T*_min_ = 0.78, *T*_max_ = 0.99	*l* = −16→16
6215 measured reflections	

### Refinement

**Table:**

Refinement on *F*^2^	Primary atom site location: structure-invariant direct methods
Least-squares matrix: full	Secondary atom site location: difference Fourier map
*R*[*F*^2^ > 2σ(*F*^2^)] = 0.057	Hydrogen site location: mixed
*wR*(*F*^2^) = 0.151	H atoms treated by a mixture of independent and constrained refinement
*S* = 1.03	*w* = 1/[σ^2^(*F*_o_^2^) + (0.0739*P*)^2^ + 0.065*P*] where *P* = (*F*_o_^2^ + 2*F*_c_^2^)/3
3039 reflections	(Δ/σ)_max_ < 0.001
235 parameters	Δρ_max_ = 0.18 e Å^−^^3^
0 restraints	Δρ_min_ = −0.22 e Å^−^^3^

### Special details

**Table:**

Geometry. All e.s.d.'s (except the e.s.d. in the dihedral angle between two l.s. planes) are estimated using the full covariance matrix. The cell e.s.d.'s are taken into account individually in the estimation of e.s.d.'s in distances, angles and torsion angles; correlations between e.s.d.'s in cell parameters are only used when they are defined by crystal symmetry. An approximate (isotropic) treatment of cell e.s.d.'s is used for estimating e.s.d.'s involving l.s. planes.
Refinement. Refinement of *F*^2^ against ALL reflections. The weighted *R*-factor *wR* and goodness of fit *S* are based on *F*^2^, conventional *R*-factors *R* are based on *F*, with *F* set to zero for negative *F*^2^. The threshold expression of *F*^2^ > σ(*F*^2^) is used only for calculating *R*-factors(gt) *etc*. and is not relevant to the choice of reflections for refinement. *R*-factors based on *F*^2^ are statistically about twice as large as those based on *F*, and *R*- factors based on ALL data will be even larger. H-atoms attached to carbon were placed in calculated positions (C—H = 0.95 − 1.00 Å). All were included as riding contributions with isotropic displacement parameters 1.2 − 1.5 times those of the attached atoms.

### Fractional atomic coordinates and isotropic or equivalent isotropic displacement parameters (Å^2^)

**Table:**

	*x*	*y*	*z*	*U*_iso_*/*U*_eq_	
O1	0.3785 (2)	0.96970 (14)	0.76705 (13)	0.0403 (4)	
O2	0.3737 (3)	0.29798 (15)	0.88255 (14)	0.0498 (5)	
N1	1.0346 (3)	0.8671 (2)	0.90170 (17)	0.0471 (5)	
N2	0.6393 (3)	1.08806 (19)	0.90280 (19)	0.0475 (6)	
H2A	0.741 (5)	1.101 (3)	0.966 (2)	0.060 (8)*	
H2B	0.559 (5)	1.159 (3)	0.909 (2)	0.064 (8)*	
C1	0.5666 (3)	0.9677 (2)	0.82491 (19)	0.0368 (5)	
C2	0.6619 (3)	0.8565 (2)	0.80085 (18)	0.0357 (5)	
C3	0.5665 (3)	0.7233 (2)	0.71189 (18)	0.0369 (5)	
H3	0.6756	0.6971	0.6629	0.044*	
C4	0.3733 (3)	0.7425 (2)	0.64460 (18)	0.0369 (5)	
C5	0.2723 (4)	0.6386 (2)	0.5477 (2)	0.0438 (6)	
H5	0.3325	0.5587	0.5229	0.053*	
C6	0.0909 (4)	0.6488 (2)	0.4883 (2)	0.0451 (6)	
H6	0.0290	0.5778	0.4223	0.054*	
C7	−0.0054 (4)	0.7651 (2)	0.52499 (19)	0.0395 (5)	
C8	−0.2030 (4)	0.7755 (3)	0.4698 (2)	0.0483 (6)	
H8	−0.2732	0.7031	0.4063	0.058*	
C9	−0.2917 (4)	0.8878 (3)	0.5073 (2)	0.0527 (7)	
H9	−0.4237	0.8935	0.4697	0.063*	
C10	−0.1911 (4)	0.9956 (3)	0.6005 (2)	0.0512 (6)	
H10	−0.2559	1.0735	0.6256	0.061*	
C11	−0.0004 (4)	0.9904 (2)	0.6562 (2)	0.0437 (6)	
H11	0.0674	1.0646	0.7189	0.052*	
C12	0.0955 (3)	0.8731 (2)	0.61949 (19)	0.0372 (5)	
C13	0.2878 (3)	0.8580 (2)	0.67621 (18)	0.0356 (5)	
C14	0.8674 (4)	0.8653 (2)	0.85785 (19)	0.0387 (5)	
C15	0.5109 (3)	0.6109 (2)	0.75824 (18)	0.0353 (5)	
C16	0.3363 (4)	0.6046 (2)	0.8117 (2)	0.0414 (6)	
H16	0.2487	0.6720	0.8190	0.050*	
C17	0.2843 (4)	0.5033 (2)	0.8551 (2)	0.0438 (6)	
H17	0.1631	0.5017	0.8914	0.053*	
C18	0.4117 (4)	0.4042 (2)	0.84493 (19)	0.0395 (5)	
C19	0.5888 (4)	0.4095 (2)	0.79246 (19)	0.0417 (6)	
H19	0.6772	0.3426	0.7858	0.050*	
C20	0.6375 (4)	0.5111 (2)	0.74989 (19)	0.0400 (5)	
H20	0.7595	0.5132	0.7142	0.048*	
C21	0.1919 (5)	0.2878 (3)	0.9351 (2)	0.0562 (7)	
H21A	0.2068	0.3672	1.0013	0.084*	
H21B	0.1803	0.2057	0.9547	0.084*	
H21C	0.0631	0.2833	0.8855	0.084*	

### Atomic displacement parameters (Å^2^)

**Table:**

	*U*^11^	*U*^22^	*U*^33^	*U*^12^	*U*^13^	*U*^23^
O1	0.0357 (8)	0.0340 (8)	0.0500 (10)	0.0045 (6)	−0.0064 (7)	0.0171 (7)
O2	0.0608 (11)	0.0361 (8)	0.0593 (11)	0.0072 (7)	0.0046 (9)	0.0271 (8)
N1	0.0425 (12)	0.0469 (11)	0.0494 (13)	0.0127 (9)	−0.0028 (10)	0.0141 (10)
N2	0.0478 (13)	0.0318 (10)	0.0568 (14)	0.0053 (8)	−0.0132 (10)	0.0138 (10)
C1	0.0327 (12)	0.0353 (11)	0.0452 (13)	0.0006 (8)	−0.0021 (10)	0.0221 (10)
C2	0.0317 (12)	0.0361 (11)	0.0414 (13)	0.0037 (8)	0.0003 (9)	0.0183 (10)
C3	0.0355 (12)	0.0365 (11)	0.0411 (13)	0.0061 (9)	0.0043 (10)	0.0169 (10)
C4	0.0357 (12)	0.0369 (11)	0.0407 (13)	0.0021 (9)	0.0034 (9)	0.0190 (10)
C5	0.0462 (14)	0.0405 (12)	0.0448 (14)	0.0063 (10)	0.0045 (11)	0.0159 (11)
C6	0.0452 (14)	0.0477 (13)	0.0397 (13)	−0.0015 (10)	−0.0010 (11)	0.0173 (11)
C7	0.0363 (12)	0.0440 (12)	0.0425 (13)	−0.0015 (9)	0.0013 (10)	0.0252 (11)
C8	0.0405 (14)	0.0583 (15)	0.0473 (15)	−0.0045 (11)	−0.0047 (11)	0.0282 (12)
C9	0.0385 (14)	0.0650 (16)	0.0605 (17)	0.0047 (11)	−0.0042 (12)	0.0349 (14)
C10	0.0418 (14)	0.0567 (15)	0.0630 (17)	0.0120 (11)	0.0006 (12)	0.0320 (14)
C11	0.0429 (13)	0.0421 (12)	0.0507 (14)	0.0055 (10)	0.0009 (11)	0.0246 (11)
C12	0.0340 (12)	0.0415 (12)	0.0420 (13)	0.0010 (9)	0.0025 (10)	0.0254 (10)
C13	0.0344 (12)	0.0351 (11)	0.0389 (12)	−0.0012 (8)	0.0003 (9)	0.0191 (10)
C14	0.0416 (14)	0.0333 (11)	0.0424 (13)	0.0069 (9)	0.0037 (10)	0.0152 (10)
C15	0.0386 (12)	0.0305 (10)	0.0372 (12)	0.0071 (8)	0.0010 (9)	0.0130 (9)
C16	0.0448 (14)	0.0347 (11)	0.0521 (15)	0.0147 (9)	0.0132 (11)	0.0199 (11)
C17	0.0476 (14)	0.0364 (12)	0.0522 (15)	0.0098 (10)	0.0119 (11)	0.0195 (11)
C18	0.0495 (14)	0.0282 (10)	0.0398 (13)	0.0029 (9)	−0.0034 (10)	0.0148 (10)
C19	0.0495 (14)	0.0322 (11)	0.0450 (14)	0.0134 (9)	0.0011 (11)	0.0147 (10)
C20	0.0381 (13)	0.0388 (12)	0.0429 (13)	0.0090 (9)	0.0045 (10)	0.0131 (10)
C21	0.0716 (18)	0.0427 (13)	0.0585 (17)	0.0007 (12)	0.0089 (14)	0.0269 (13)

### Geometric parameters (Å, º)

**Table:**

O1—C1	1.359 (3)	C8—C9	1.355 (4)
O1—C13	1.387 (3)	C8—H8	0.9500
O2—C18	1.372 (3)	C9—C10	1.399 (4)
O2—C21	1.418 (3)	C9—H9	0.9500
N1—C14	1.154 (3)	C10—C11	1.370 (3)
N2—C1	1.339 (3)	C10—H10	0.9500
N2—H2A	0.96 (3)	C11—C12	1.423 (3)
N2—H2B	0.95 (3)	C11—H11	0.9500
C1—C2	1.359 (3)	C12—C13	1.422 (3)
C2—C14	1.417 (3)	C15—C16	1.381 (3)
C2—C3	1.513 (3)	C15—C20	1.396 (3)
C3—C4	1.516 (3)	C16—C17	1.387 (3)
C3—C15	1.522 (3)	C16—H16	0.9500
C3—H3	1.0000	C17—C18	1.391 (3)
C4—C13	1.365 (3)	C17—H17	0.9500
C4—C5	1.409 (3)	C18—C19	1.386 (3)
C5—C6	1.363 (3)	C19—C20	1.380 (3)
C5—H5	0.9500	C19—H19	0.9500
C6—C7	1.415 (3)	C20—H20	0.9500
C6—H6	0.9500	C21—H21A	0.9800
C7—C12	1.409 (3)	C21—H21B	0.9800
C7—C8	1.423 (3)	C21—H21C	0.9800
			
C1—O1—C13	119.06 (17)	C11—C10—H10	119.6
C18—O2—C21	117.97 (18)	C9—C10—H10	119.6
C1—N2—H2A	123.6 (16)	C10—C11—C12	119.6 (2)
C1—N2—H2B	119.0 (17)	C10—C11—H11	120.2
H2A—N2—H2B	115 (2)	C12—C11—H11	120.2
N2—C1—C2	127.4 (2)	C7—C12—C13	118.0 (2)
N2—C1—O1	110.53 (18)	C7—C12—C11	119.3 (2)
C2—C1—O1	122.1 (2)	C13—C12—C11	122.6 (2)
C1—C2—C14	119.1 (2)	C4—C13—O1	123.04 (19)
C1—C2—C3	123.89 (19)	C4—C13—C12	122.7 (2)
C14—C2—C3	116.96 (18)	O1—C13—C12	114.30 (18)
C2—C3—C4	109.13 (17)	N1—C14—C2	177.3 (2)
C2—C3—C15	111.84 (18)	C16—C15—C20	117.4 (2)
C4—C3—C15	111.76 (18)	C16—C15—C3	121.60 (18)
C2—C3—H3	108.0	C20—C15—C3	121.0 (2)
C4—C3—H3	108.0	C15—C16—C17	122.3 (2)
C15—C3—H3	108.0	C15—C16—H16	118.8
C13—C4—C5	117.7 (2)	C17—C16—H16	118.8
C13—C4—C3	121.9 (2)	C16—C17—C18	119.3 (2)
C5—C4—C3	120.39 (19)	C16—C17—H17	120.4
C6—C5—C4	122.1 (2)	C18—C17—H17	120.4
C6—C5—H5	118.9	O2—C18—C19	116.25 (19)
C4—C5—H5	118.9	O2—C18—C17	124.5 (2)
C5—C6—C7	120.1 (2)	C19—C18—C17	119.3 (2)
C5—C6—H6	120.0	C20—C19—C18	120.5 (2)
C7—C6—H6	120.0	C20—C19—H19	119.8
C12—C7—C6	119.2 (2)	C18—C19—H19	119.8
C12—C7—C8	118.9 (2)	C19—C20—C15	121.3 (2)
C6—C7—C8	121.8 (2)	C19—C20—H20	119.4
C9—C8—C7	120.4 (2)	C15—C20—H20	119.4
C9—C8—H8	119.8	O2—C21—H21A	109.5
C7—C8—H8	119.8	O2—C21—H21B	109.5
C8—C9—C10	120.8 (2)	H21A—C21—H21B	109.5
C8—C9—H9	119.6	O2—C21—H21C	109.5
C10—C9—H9	119.6	H21A—C21—H21C	109.5
C11—C10—C9	120.9 (2)	H21B—C21—H21C	109.5
			
C13—O1—C1—N2	174.13 (19)	C10—C11—C12—C7	1.2 (3)
C13—O1—C1—C2	−4.3 (3)	C10—C11—C12—C13	−176.9 (2)
N2—C1—C2—C14	−4.0 (4)	C5—C4—C13—O1	−176.9 (2)
O1—C1—C2—C14	174.2 (2)	C3—C4—C13—O1	4.8 (3)
N2—C1—C2—C3	179.2 (2)	C5—C4—C13—C12	4.2 (3)
O1—C1—C2—C3	−2.6 (3)	C3—C4—C13—C12	−174.2 (2)
C1—C2—C3—C4	9.3 (3)	C1—O1—C13—C4	3.3 (3)
C14—C2—C3—C4	−167.6 (2)	C1—O1—C13—C12	−177.71 (19)
C1—C2—C3—C15	−114.9 (2)	C7—C12—C13—C4	−2.0 (3)
C14—C2—C3—C15	68.2 (3)	C11—C12—C13—C4	176.1 (2)
C2—C3—C4—C13	−10.2 (3)	C7—C12—C13—O1	178.94 (18)
C15—C3—C4—C13	114.1 (2)	C11—C12—C13—O1	−2.9 (3)
C2—C3—C4—C5	171.5 (2)	C2—C3—C15—C16	74.0 (3)
C15—C3—C4—C5	−64.3 (3)	C4—C3—C15—C16	−48.7 (3)
C13—C4—C5—C6	−2.3 (3)	C2—C3—C15—C20	−104.9 (2)
C3—C4—C5—C6	176.1 (2)	C4—C3—C15—C20	132.4 (2)
C4—C5—C6—C7	−1.7 (4)	C20—C15—C16—C17	−0.6 (3)
C5—C6—C7—C12	3.9 (3)	C3—C15—C16—C17	−179.5 (2)
C5—C6—C7—C8	−175.3 (2)	C15—C16—C17—C18	0.0 (4)
C12—C7—C8—C9	0.4 (3)	C21—O2—C18—C19	−178.9 (2)
C6—C7—C8—C9	179.6 (2)	C21—O2—C18—C17	0.3 (3)
C7—C8—C9—C10	0.1 (4)	C16—C17—C18—O2	−178.6 (2)
C8—C9—C10—C11	0.1 (4)	C16—C17—C18—C19	0.6 (3)
C9—C10—C11—C12	−0.8 (4)	O2—C18—C19—C20	178.7 (2)
C6—C7—C12—C13	−2.1 (3)	C17—C18—C19—C20	−0.6 (3)
C8—C7—C12—C13	177.1 (2)	C18—C19—C20—C15	0.0 (3)
C6—C7—C12—C11	179.7 (2)	C16—C15—C20—C19	0.6 (3)
C8—C7—C12—C11	−1.1 (3)	C3—C15—C20—C19	179.5 (2)

### Hydrogen-bond geometry (Å, º)

**Table:**

*D*—H···*A*	*D*—H	H···*A*	*D*···*A*	*D*—H···*A*
N2—H2*A*···N1^i^	0.96 (3)	2.03 (3)	2.995 (3)	178 (3)
N2—H2*B*···O2^ii^	0.95 (3)	2.10 (3)	3.028 (3)	166 (2)

Symmetry codes: (i) −*x*+2, −*y*+2, −*z*+2; (ii) *x*, *y*+1, *z*.
