# Supplementary figures and images for: Crystal structure of 2-amino-4-(4-meth­oxy­phen­yl)-4H-benzo[g]chromene-3-carbo­nitrile
Source: Acta Crystallogr E Crystallogr Commun. 2015 Dec 6;71(Pt 12):o1017–8. doi: 10.1107/S205698901502280X (PMC4719954; doi:10.1107/S205698901502280X)

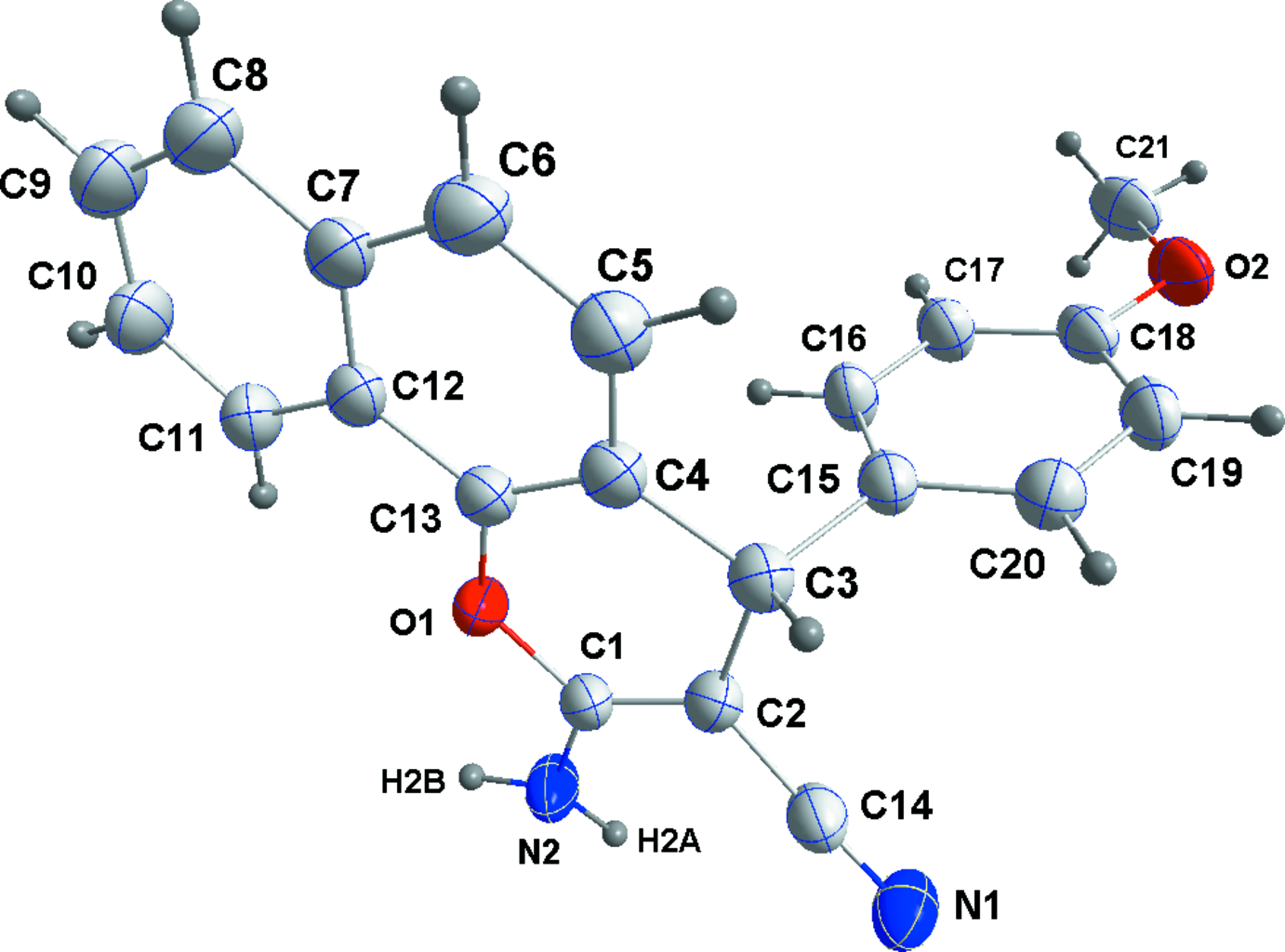

Supplement: Supplementary file 4 [file e-71-o1017-fig1.tif]

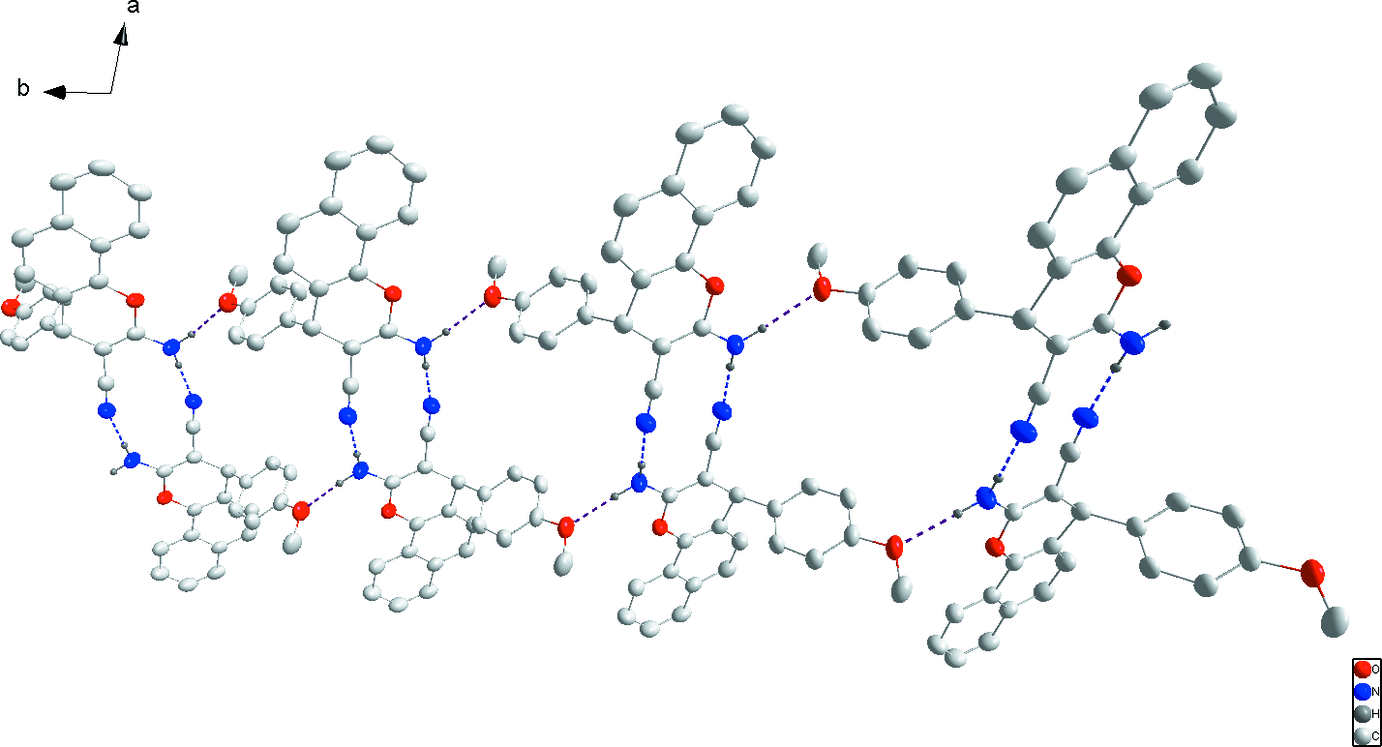

Supplement: Supplementary file 5 [file e-71-o1017-fig2.tif]

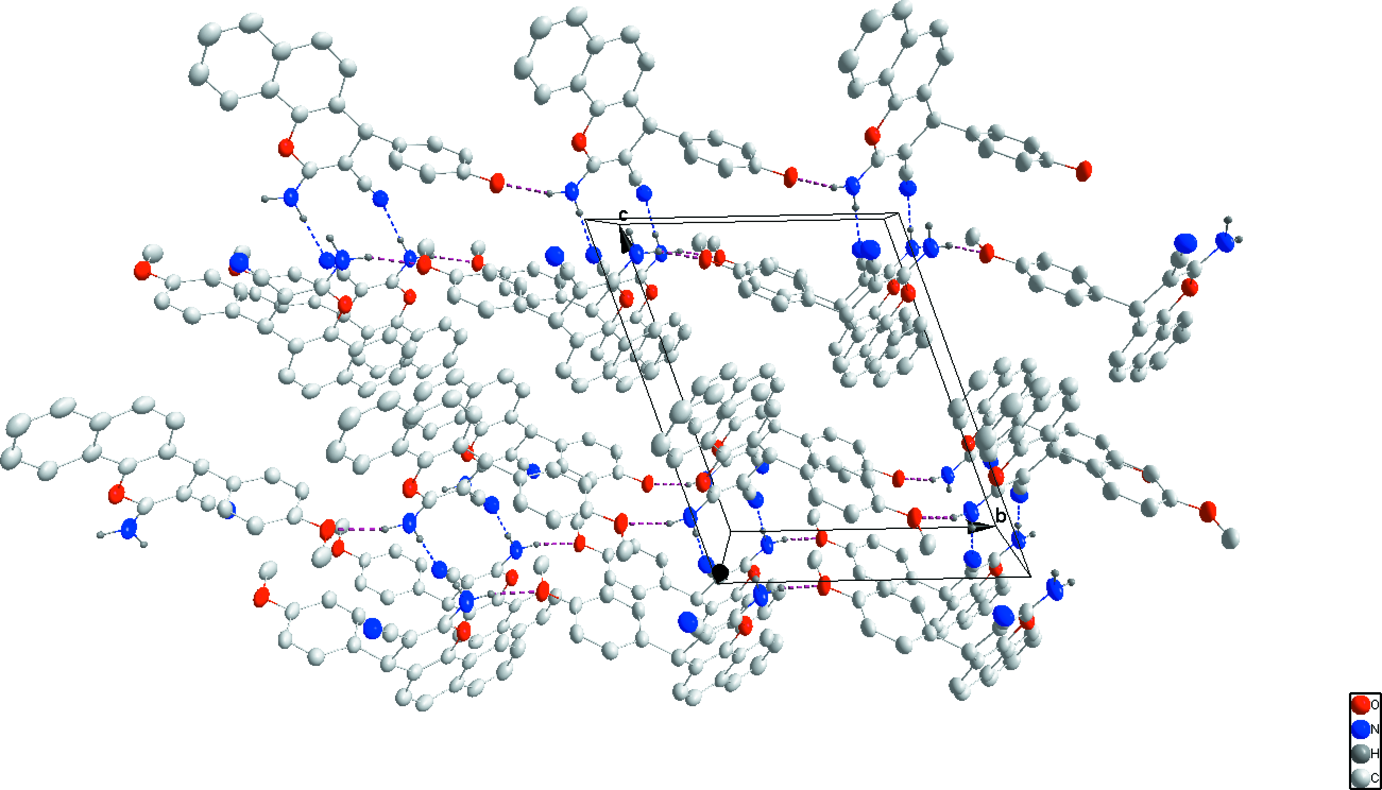

Supplement: Supplementary file 6 [file e-71-o1017-fig3.tif]
